# Determining the difference in the efficacy and safety of self-expandable metallic stents as a bridge to surgery for obstructive colon cancer among patients in the CROSS 0 group and those in the CROSS 1 or 2 group: a pooled analysis of data from two Japanese prospective multicenter trials

**DOI:** 10.1007/s00595-020-01970-3

**Published:** 2020-02-06

**Authors:** Takeshi Ohki, Shuntaro Yoshida, Masakazu Yamamoto, Hiroyuki Isayama, Tomonori Yamada, Takeaki Matsuzawa, Shuji Saito, Toshio Kuwai, Masafumi Tomita, Toshiyasu Shiratori, Mamoru Shimada, Tomio Hirakawa, Koichi Koizumi, Yoshihisa Saida

**Affiliations:** 1 Department of Surgery, Institute of Gastroenterology, Tokyo Women’s Medical University, 8-1 Kawada-cho, Shinjuku-ku, Tokyo, 162-8666 Japan; 2 Japan Colonic Stent Safe Procedure Research Group, Tokyo, Japan; 3 Department of Endoscopy and Endoscopic Surgery, Graduate School of Medicine, The University of Tokyo, Tokyo, Japan; 4 Department of Gastroenterology, Graduate School of Medicine, Juntendo University, Tokyo, Japan; 5 Department of Gastroenterology, Japanese Red Cross Nagoya Daini Hospital, Nagoya, Japan; 6 Department of Surgery, IMS Miyoshi General Hospital, Miyoshi, Japan; 7 Division of Surgery, Gastrointestinal Center, Yokohama Shin-Midori General Hospital, Yokohama, Japan; 8 Department of Gastroenterology, National Hospital Organization Kure Medical Center and Chugoku Cancer Center, Kure, Japan; 9 Department of Surgery, Kishiwada Tokushukai Hospital, Osaka, Japan; 10 Department of Gastroenterology, Kameda Medical Center, Kamogawa, Japan; 11 Department of Surgery, Toyonaka Midorigaoka Hospital, Osaka, Japan; 12 Department of Gastroenterology, Toyonaka Midorigaoka Hospital, Osaka, Japan; 13 Department of Gastroenterology, Tokyo Metropolitan Cancer and Infectious Disease Center Komagome Hospital, Tokyo, Japan; 14 Department of Surgery, Toho University Ohashi Medical Center, Tokyo, Japan

**Keywords:** Colorectal cancer, Colorectal obstruction, Bridge to surgery, Self-expandable metallic stents, Pooled analysis

## Abstract

**Purpose:**

This study compared the feasibility and safety of endoscopic placement of self-expandable metallic stents (SEMSs) as a bridge to surgery (BTS) between patients with obstructive colorectal cancer (CRC) classified as ColoRectal Obstruction Scoring System (CROSS) 0 and those with CROSS 1 or 2.

**Methods:**

We conducted a post hoc analysis of two prospective, observational, single-arm multicenter clinical trials and performed a pooled analysis of the data. In total, 336 consecutive patients with malignant colorectal obstruction underwent SEMS placement. The primary endpoint was clinical success, defined as resolution of symptoms and radiological findings within 24 h. Secondary endpoints were technical success and adverse events.

**Results:**

High clinical (98.0% vs. 98.4%) and technical (96.7% vs. 97.8%) success rates were observed in both groups (CROSS 0 vs. CROSS 1 or 2). The adverse event rate was low. The mean stricture length was lower (3.8 ± 1.2 cm vs. 4.4 ± 1.8 cm) and laparoscopic surgery more common (56.7% vs 52.2%) in the CROSS 0 group than in the CROSS 1 and 2 group.

**Conclusion:**

This study was the first to compare the degree of stricture in different CROSS groups and demonstrated comparable results with respect to the short-term efficacy and safety of SEMS placement as a BTS for obstructive CRC in CROSS 0, 1, and 2 patients.

## Introduction

Endoscopic placement of self-expandable metal stents (SEMSs) has two major indications: to relieve the symptoms of bowel obstruction and restore the bowel function in a palliative setting and to achieve bowel decompression as a bridge to surgery (BTS). Therefore, when patients with colorectal cancer (CRC) with acute obstruction undergo radical resection, they need a diverting stoma to reduce the risk of anastomotic leakage. Recently, with the advent of SEMS placement before radical surgery, stomas have not necessarily been required [1].

Clinical guidelines from the European Society of Gastrointestinal Endoscopy (ESGE) state that SEMSs for BTS may not be safe in colonic obstruction associated with malignancy, especially on the left side of the colon [2]. This recommendation is based on the findings of various randomized controlled trials (RCTs) and cohort studies indicating that the success rate of using a SEMS as BTS was as low as 47% (47–100%), while the perforation rate was as high as 8.7% (0–12.8%) [3–11]. Therefore, SEMS placement for oncological indications appears to affect the long-term prognosis.

In Japan, SEMS placement has been covered by national health insurance since 2012. The Japan Colonic Stent Safety Procedure Research Group (JCSSPRG) was founded with the sole purpose of ensuring the safe use of SEMSs in Japan. Our organization began our efforts by modifying the scoring system for the assessment of the alimentation status of patients with malignant gastric outlet obstruction [12] to include clinical features of obstruction along with the oral intake status. This modified system is called the ColoRectal Obstruction Scoring System (CROSS) and uses a point score system, as shown in Table 1. CROSS 0 patients need emergency surgery or SEMS placement. CROSS 1 or 2 patients are candidates for elective surgery. CROSS 3 and 4 patients can receive food; therefore, SEMS placement is not necessary. The CROSS is widely accepted for the evaluation of the severity of obstruction in Japan. In simple terms, colonic stenting for CROSS 0 patients seems to be more difficult than that for CROSS 1 or 2 patients. Thus far, however, there has been no study comparing the effectiveness and safety of SEMS placement in the CROSS 1 or 2 group and CROSS 0 group.

**Table 1:** The ColoRectal obstruction scoring system

Level of oral intake	Score
Requiring continuous decompression	0
No oral intake	1
Liquid or enteral nutrient intake	2
Soft solids, low-residue, and full diet with symptoms of stricture^a^	3
Soft solids, low-residue, and full diet without symptoms of stricture^a^	4

^a^Symptoms of stricture include abdominal pain/cramps, abdominal distension, nausea, vomiting, constipation, and diarrhea, which are related to gastrointestinal transit

Our group recently launched a phase III RCT called the colonic stent for “Bridge to Surgery” prospective randomized controlled trial (COBRA). We compared this method to the treatment with non-stenting surgery in stage II/III obstructive colon cancer in order to investigate whether or not decompression by colonic stenting as a BTS for obstructive CRC is non-inferior to surgery (considered the standard treatment) [13]. This trial was registered with the University Hospital Medical Information Network (UMIN) Clinical Trial Registry (UMIN-CTR000026158), and the long-term prognosis is assessed in terms of the three-year disease-free survival (DFS), which is an index of the oncological outcome of CRC after curative resection. Of note, however, this trial only includes patients with obstructive CRC in CROSS 1 and 2 categories.

To extrapolate the results of this study to CROSS 0 patients as well, the present study aimed to clarify whether or not the effectiveness and safety of SEMS placement as a BTS in the CROSS 0 group were comparable to those in the CROSS 1 or 2 group.

## Methods

### Study design and population

A post hoc analysis of two prospective, observational, single-arm multicenter clinical trials involving SEMSs were carried out. One clinical trial registered with the University Hospital Medical Information Network Clinical Trial Registry (UMIN000007953) was conducted from June 2012 to October 2013 using the WallFlex colonic stent (Boston Scientific, Natick, MA, USA). The other clinical trial (UMIN000011304) was conducted from October 2013 to May 2014 using the Niti-S colonic stent (Taewoong Medical, Gimpo-si, Gyeonggi-do, South Korea).

Before these trials began, a website (https://colon-stent.com/) with information regarding the standard methods of SEMS placement based on previously published data was launched. A workshop was conducted to develop guidelines for a standard, adequate, and safe SEMS placement procedure. More than 140 physicians across Japan participated, and several of them offered their experiences to this end. This was followed by discussions among physicians at the participating facilities. The core points of these discussions were consolidated into a brief guideline that was uploaded to the website.

Patients with acute colorectal obstruction or symptomatic strictures secondary to malignant neoplasms were enrolled at 43 participating facilities (14 academic centers and 29 community hospitals) under these two trials. Approval was obtained from the respective institutional review boards of each participating facility before the start of the trials. At the time of patient enrollment, the treatment objective (BTS or palliative) was determined based on the stage of malignant disease, coexisting illness, age, and (in some cases) patient choice.

### Exclusion criteria and endpoints

Cases with previous colonic SEMS placement, enteral ischemia, suspected or impending perforation, intra-abdominal abscess, contraindications to endoscopy, and the use of a SEMS for indications other than those outlined under the trial were excluded. Patients were followed for a period of seven days.

Clinical success was the primary endpoint of this study, defined as an improvement in obstructive symptoms resulting in the resumption of food intake within 24 h of SEMS placement. Technical success was the secondary endpoint, defined as successful deployment of a SEMS to cover the full length of the stricture on the first attempt with no adverse events. The clinical success of BTS was defined as improvement in obstructive symptoms before surgery without any stent-related adverse events or need for endoscopic re-intervention or emergency surgery.

Adverse events included stent migration, perforation, bleeding, a fever, abdominal pain, tenesmus, and primary anastomosis with or without a diverting stoma. Complete obstruction was diagnosed when any of the following was present: inability to pass flatus, lack of water-soluble contrast passing proximal to the lesion on contrast enema, or lack of an endoscopically visible proximal lumen on colonoscopy [14–16].

### Type of SEMSs and the placement procedure

Patients were treated using either an uncovered WallFlex colonic stent or a Nits-S colonic stent. WallFlex stents are available in 2 external diameters (22 and 25 mm) in 3 different lengths (6, 9, and 12 cm). Niti-S stents are also available in 2 external diameters (18 and 22 mm) and 4 different lengths (6, 8, 10, and 12 cm).

The procedure for SEMS placement was as follows: a guidewire was used to establish access through the stricture, and a contrast tube was inserted into the proximal lumen. Fluoroscopy was used to measure the length of the stricture and determine the number of stents required to cross the stricture. Intraluminal or extraluminal marking using an endoscopic clip, lipoid, or radiopaque marker was performed as instructed by the endoscopist in order to locate the stricture. No dilation of the stricture was done before SEMS placement.

### Statistical analyses

All analyses were performed using the JMP software program (ver. 12.2.0; SAS Institute, Chicago, IL, USA). Continuous variables are expressed as the means and standard deviations (SD), while nominal variables are described using numbers and percentages. Continuous variables were compared using the Kruskal–Wallis test, and nominal variables were compared using the Chi-squared test. A *P* value of < 0.05 was considered significant. Multivariate analyses were performed to study the effects of various risk factors on clinical failure.

## Results

**Fig. 1:**
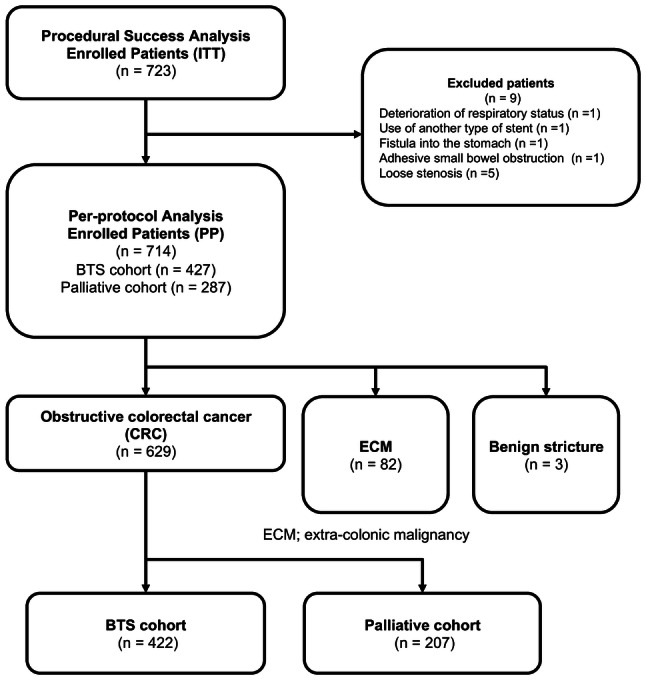
Flowchart of the study showing the number of patients enrolled who had obstructive CRC. *BTS* bridge to surgery, *CRC* colorectal cancer, *ECM* extra-colonic malignancy, *ITT* intention to treat, *PP* per-protocol

Of the 723 consecutive patients enrolled in the study, 9 were excluded due to deterioration of respiratory status (*n* = 1), usage of another type of SEMS (*n* = 1), fistula into the stomach (*n* = 1), adhesive small bowel obstruction (*n* = 1), and loose stenosis (*n* = 5) (Fig. 1). Thus, the per-protocol cohort comprised the remaining 714 patients.

Of these patients, 629 had obstructive CRC, 82 had extra-colonic malignancy (ECM), and 3 had benign stricture. Of the 629 patients with obstructive CRC, the treatment objective was BTS in 422 (62.1%) patients and palliation in the remaining 207 (32.9%) patients.

**Fig. 2:**
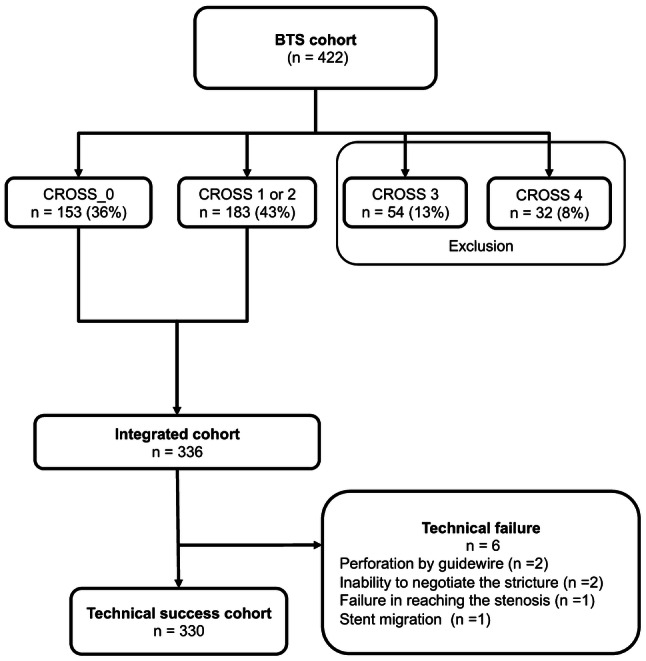
Study profile showing the number of patients in each CROSS group and the derivation of the technical success cohort. *CROSS* ColoRectal Obstruction Scoring System

Of the 422 BTS patients, 153 (36%) were graded as CROSS 0, 183 (43%) as CROSS 1 or 2, 54 (13%) as CROSS 3, and 32 (8%) as CROSS 4. Of these, CROSS 3 and 4 patients were excluded from the analysis as shown in Fig. 2. Thus, the “integrated cohort” comprised 336 patients. To evaluate adverse events after SEMS placement, all but six technically unsuccessful cases were defined as the “technical success cohort” in Fig. 2.

### Patient characteristics

Table 2 shows the characteristics and details of the clinical presentation of the patients included in the study. The mean age of the patients was 69.7 years old, and male patients comprised 57.7% of the cohort (*n* = 194). Symptoms of obstruction were present in 334 (99.4%) patients and included a deteriorating defecatory pattern (94.3%), bloating (88.1%), abdominal pain or cramps (81.0%), and nausea or vomiting (54.2%). Of the 336 patients, 334 (99.4%) had primary CRC, 1 had dissemination of CRC, and 1 had local recurrence of CRC. Left-sided obstruction was more common than right-sided (241, 71.7%), and the sigmoid colon was the most common site of obstruction (106, 31.5%). Other associated conditions were localized tumor (246, 73.2%), liver metastasis (52, 15.5%), lung metastasis (22, 6.5%), peritoneal carcinomatosis (31, 9.2%), and other metastases (48, 14.3%). Ascites was present in 68 (20.2%) patients. Complete obstruction was present in 303 (90.2%) patients. All but 1 had a single obstruction (442, 99.7%). The mean stricture length was 4.1 cm. The classification of patients on the basis of their Eastern Cooperative Oncology Group (ECOG) performance status (PS) was as follows: 135 (40.1%) patients were classified as PS 0, 153 (45.5%) as PS 1, 21 (6.3%) as PS 2, 21(6.3%) as PS 3, and 6 (1.8%) as PS 4. Regarding the American Society of Anesthesiologists (ASA) physical status classification of patients, 181 (53.9%) patients were classified as ASA 1, 133 (39.6%) as ASA 2, 20 (6.0%) as ASA 3, and 2 (0.5%) as ASA 4. Fifteen patients (5%) underwent chemotherapy before SEMS placement.

**Table 2:** Characteristics and details of clinical presentation of patients included in the “integrated cohort” (*n* = 336)

Patient characteristics	
Age, years	69.7 ± 11.6
Sex, male/female	194/142, (57.7/42.3)
Symptoms of obstruction present	334 (99.4)
Deteriorating defecatory pattern	317 (94.3)
Bloating	296 (88.1)
Abdominal pain/cramps	281 (83.6)
Nausea/vomiting	182 (54.2)
Tumor origin	
Primary colorectal cancer (CRC)	334 (99.4)
Dissemination of CRC	1 (0.3)
Local recurrence of CRC	1 (0.3)
Site of obstruction, left/right	241/95, (71.7/28.3)
Tumor localization (*n* = 338)	
Rectum	10 (3.0)
Rectosigmoid junction	36 (10.7)
Sigmoid colon^a^	106 (31.5)
Sigmoid-descending colon junction	33 (9.8)
Descending colon^a^	59 (17.6)
Splenic flexure	14 (4.2)
Transverse colon	43 (12.8)
Hepatic flexure	9 (2.7)
Ascending colon	17 (5.1)
Cecum	11 (3.3)
Associated conditions	
Localized tumor	246 (73.2)
Liver metastasis	53 (15.8)
Lung metastasis	22 (6.5)
Peritoneal carcinomatosis	31 (9.2)
Other metastases	48 (14.3)
Ascites	68 (20.2)
Complete obstruction^b^	303 (90.2)
Number of sites of obstruction	
1	335 (99.7)
2	1 (0.3)
Stricture length, cm	4.1 ± 1.6
ECOG performance status	
0	135 (40.1)
1	153 (45.5)
2	21 (6.3)
3	21 (6.3)
4	6 (1.8)
ASA performance status	
1	181 (53.9)
2	133 (39.5)
3	20 (6.0)
4	2 (0.6)
5	0 (0)
CROSS	
0	153 (45.5)
1	127 (37.8)
2	56 (16.7)
Treatment history	
Chemotherapy	15 (5)
Radiotherapy	0 (0)

Values are the mean ± standard deviation or n (%)

*ASA* American Society of Anesthesiologists, *CROSS* ColoRectal Obstruction Scoring System, *ECOG* Eastern Cooperative Oncology Group

^a^One patient had two obstruction sites at the sigmoid and descending colon. The other patient had a long obstruction from the sigmoid-descending junction to the descending colon

^b^Complete obstruction was diagnosed when any of the following was present: inability to pass flatus, lack of water-soluble contrast passing proximal to the lesion, and lack of an endoscopically visible proximal lumen

### Interventions before SEMS placement and procedural details

Digestive tract decompression using a tube was performed in 98 patients (29.2%) at the time of the diagnosis (Table 3), while stricture balloon dilation was performed in 6 (1.8%) patients. As preparation for SEMS placement, 114 patients (33.9%) were administered cleansing enema. Intraluminal and/or extraluminal stricture marking was done in 241 (71.7%) patients. The technical failure rate (where an SEMS could not be placed in a stricture) was 1.9% (*n* = 6). Of the remaining 330 patients in whom stents were successfully deployed, 322 (97.6%) had a single stricture with 1 stent, 7 (2.1%) had a single stricture with 2 stents, and 1 (0.3%) had a double stricture with 2 stents. In total, 338 stents were deployed, of which WallFlex colonic stents constituted 73.4% (*n* = 248). The 22 mm stents were the most commonly used stents (309, 91.4%). The mean procedure time for stent placement was 37.9 min.

**Table 3:** Interventions before SEMS placement and procedural details of patients included in the “integrated cohort” (*n* = 336)

Interventions before SEMS placement	
Digestive tract decompression before SEMS placement	98 (29.2)
Naso-gastric tube	22 (6.5)
Naso-intestinal tube	38 (11.3)
Trans-anal tube	41 (12.2)
Stricture balloon dilation	6 (1.8)
Preparation for SEMS placement	
Cleansing enema	114 (33.9)
Oral bowel cleansing	16 (4.8)
Stricture marking done	241 (71.7)
Intraluminal	221 (65.8)
Extraluminal	25 (7.4)
Details of SEMS procedure	
Strictures and stents placed	
Stricture with no stent (technical failure)	6 (1.9)
Single stricture with 1 stent	322/330 (97.6)
Single stricture with 2 stents	7/330 (2.1)
Double stricture with 2 stents	1/330 (0.3)
Stent type (total number of stents deployed, 338^a^)	
WallFlex colonic stent	248 (73.4)
Niti-S colonic stent	90 (26.6)
6 cm long	179 (53.0)
8 cm long	36 (10.7)
9 cm long	87 (25.7)
10 cm long	22 (6.5)
12 cm long	14 (4.2)
18 mm diameter	11 (3.3)
22 mm diameter	309 (91.4)
25 mm diameter	18 (5.3)
Procedure time in the technical success cohort, min	37.9 ± 20.4
Technical success rate	330 (98.2)
Clinical success rate	327 (97.3)
Clinical success rate of BTS	305 (90.8)

Values are the mean ± standard deviation or *n* (%)

*SEMS* self-expandable metallic stent, *BTS* bridge to surgery

^a^Denominator for calculating the stent percentages

The technical success rate and clinical success rate were 98.2% and 97.3%, respectively (Table 3). However, following successful stent placement, adverse events occurred in 22 patients, bringing the clinical success rate of BTS to 90.8%. Some of the adverse events observed following SEMS placement included perforation in three patients, stent migration in two patients, and stent occlusion in one patient. Perforation related to guidewire manipulation was observed in one patient in the CROSS 1 and 2 groups. There were two stent-related perforations. Perforation caused by appendicitis related to stent placement was observed in one patient in the CROSS 1 and 2 groups. Perforation at the tumor site was observed in one patient in the CROSS 0 group. No additional stent placement for migration was needed. Although stent re-obstruction caused by stool occurred in one patient in the CROSS 0 group, obstruction was treated with endoscopy.

### The comparison of CROSS 0 and CROSS 1 or 2 (Tables 4, 5, and 6)

**Table 4:** A comparison of CROSS 0 and CROSS 1 or 2 patients included in the “integrated cohort” (*n* = 336)

	CROSS 0 (*n* = 153)	CROSS 1 or 2 (*n* = 183)	*P* value
Age, years	70.1 ± 11.9	69.4 ± 11.2	0.31
Sex, male/female	94/59 (61.4/38.6)	100/83 (54.6/45.4)	0.21
Symptoms of obstruction present	153 (100)	181 (98.9)	0.19
Left-sided obstruction	105 (68.6)	136 (74.3)	0.25
Complete obstruction^a^	146 (95.4)	157 (85.8)	0.003
Localized tumor	114 (74.5)	132 (72.1)	0.62
Stricture length, cm	3.8 ± 1.2	4.4 ± 1.8	0.002
Digestive tract decompression before SEMS placement	71 (46.1)	27 (14.8)	< 0.0001
ECOG performance status			0.16
0	67 (43.8)	68 (37.2)	
1	59 (38.6)	94 (51.4)	
2	11 (7.2)	10 (5.5)	
3	13 (8.5)	8 (4.4)	
4	3 (2.0)	3 (1.6)	
ASA performance status			0.18
1	75 (49.0)	106 (57.9)	
2	70 (45.8)	63 (34.4)	
3	7 (4.6)	13 (7.1)	
4	1 (0.7)	1 (0.6)	
5	0	0	
Technical success rate	150 (98.0)	180 (98.4)	0.82
Clinical success rate of BTS	139 (90.9)	166 (90.7)	0.97
Procedural time, min	39.1 ± 21.3	37.2 ± 19.7	0.41

Values are the mean ± standard deviation or n (%)

*ASA* American Society of Anesthesiologists, *CROSS* ColoRectal Obstruction Scoring System, *ECOG* Eastern Cooperative Oncology Group, *SEMS* self-expandable metallic stent, *BTS* bridge to surgery

^a^Complete obstruction was diagnosed when any of the following was present: inability to pass flatus, lack of water-soluble contrast passing proximal to the lesion, and lack of an endoscopically visible proximal lumen

**Table 5:** A comparison of the adverse events in colonic stenting between CROSS 0 and CROSS 1 or 2 patients in the “technical success cohort” (*n* = 330)

Adverse events^a^	CROSS 0 (*n* = 150)	CROSS 1 or 2 (*n* = 180)	
Early adverse events (< 7 days)	9 (6.0)	16 (9.0)	0.32
Stent migration	0	2 (1.1)	0.20
Perforation	1 (0.7)	2 (1.1)	0.67
Stent occlusion	0	1 (0.7)	0.27
Bleeding	1 (0.7)	3 (1.7)	0.41
Fever	1 (0.7)	1 (0.6)	0.90
Abdominal pain	3 (2.0)	4 (2.2)	0.89
Tenesmus	2 (1.3)	2 (1.1)	0.85
Others	5 (3.3)	5 (2.8)	0.77

*CROSS* ColoRectal Obstruction Scoring System

^a^Patients without technical success were excluded

**Table 6:** A comparison of the surgical details, mortality, and morbidity between CROSS 0 and CROSS 1 or 2 patients in the “Technical success cohort” (*n* = 330)

	CROSS_0 (*n* = 150)	CROSS 1 or 2 (*n* = 180)	
Type of surgery			0.049
Open surgery	50 (33.3)	78 (43.3)	
Laparoscopic surgery	85 (56.7)	94 (52.2)	
Conversion	15 (10.0)	8 (4.4)	
Primary anastomosis	139 (92.7)	163 (90.6)	0.49
Primary anastomosis with diverting stoma	4 (2.7)	4 (2.2)	0.79
Time to surgery after stenting, d	20.4 ± 16.2	21.6 ± 17.0	0.51
Time to discharge after surgery, d	16.1 ± 12.0	15.8 ± 12.9	0.80
Mortality	1 (0.3)	0	0.46
Morbidity			
Bleeding	0	0	
Anastomotic leakage requiring for surgery	0	2 (1.1)	0.50
Anastomotic leakage treated by conservative therapy	7 (4.7)	7 (3.9)	0.79
Intra-abdominal abscess	1 (0.6)	6 (3.3)	0.13
Wound infection	4 (2.6)	1 (0.6)	0.18
Postoperative intestinal obstruction	2 (1.3)	0	0.21
Respiratory comorbidity	2 (1.3)	0	0.21

*CROSS* ColoRectal Obstruction Scoring System

^a^Patients without technical success were excluded

There was a statistically significant difference in the severity of stenosis (complete obstruction vs. incomplete obstruction) between the two groups, with more patients from the CROSS 0 group having complete obstruction (146 (95.4%) vs. 157 (85.8%), *P* = 0.003) (Table 4). Although high rates of complete occlusion were shown (95.4% in the CROSS 0 group and 85.8% in the CROSS 1 or 2 group), there was a statistically significant difference between the 2 cohorts. The mean stricture length was found to be significantly lower in the CROSS 0 group than in the CROSS 1 or 2 group (3.8 ± 1.2 cm vs. 4.4 ± 1.8 cm, *P* = 0.002). Digestive tract decompression before SEMS placement was performed in significantly more patients classified as CROSS 0 than in those classified as CROSS 1 or 2 (71 [46.1%] vs. 27 [14.8%], *P* < 0.0001). There was no significant difference in the technical and clinical success rates between the two groups.

The two groups were comparable with respect to the age, sex, tumor location, ECOG and ASA status, technical/clinical success rates, and procedural times, (Tables 4 and 5). Regarding the types of surgery, laparoscopic surgery was performed more frequently in the CROSS 0 group than in the CROSS 1 or 2 group (56.7% vs. 52.2%, *P* = 0.049). In addition, the primary anastomosis rates with and without diverting stoma, time to surgery, and time to discharge after surgery in both groups were comparable (Table 6). The mortality and morbidity due to surgery after stenting were also similar (Table 6).

### Factors associated with clinical failure

A multivariate analysis was performed to study the effects of various risk factors on clinical failure (Table 7); it showed that complete obstruction was an independent predictive factor of clinical success.

**Table 7:** Results of the multivariate analysis of predictive factors for clinical failure with colonic stenting for symptomatic CRC

Risk factor	Odds ratio	95% CI	*P* value
CROSS grading (CROSS 0 vs. CROSS 1 or 2)	2.19	0.40–11.97	0.37
Digestive tract decompression before SEMS placement	1.83	0.35–9.29	0.47
Complete obstruction	0.09	0.02–0.39	0.001
Stricture length (per cm)	1.30	0.83–1.03	0.26

*CRC* colorectal cancer, *CI* confidence interval, *CROSS* ColoRectal Obstruction Scoring System, *SEMS* self-expandable metallic stent

## Discussion

This study shows that SEMS placement as a BTS for obstructive CRC has good efficacy and high safety. The JCSSPRG, established in 2012 with the sole purpose of ensuring the safe use of SEMS in Japan, conducted two large multicenter, prospective studies in relation to SEMSs, the first of its kind in Japan. In 1 study, a total of 518 patients with malignant colorectal obstruction were treated with the WallFlex colonic stent, while in the other, 205 patients were treated with the Niti-S colonic stent.

Matsuzawa et al. reported the short-term outcomes in terms of the technical success rate (99.5%) and clinical success rate (97.9%) of SEMS placement using a WallFlex colonic stent with strict inclusion criteria and stricture marking [16]. Saito et al. found that placement of the WallFlex colonic stent as a BTS for malignant colorectal obstruction was a feasible approach [17]. Similarly, our group also noted good results when using a Niti-S colonic stent [18, 19]. Tomita et al. concluded that SEMS placement for malignant colorectal obstruction as a BTS was safe and effective with respect to peri-procedural outcomes using a pooled analysis of two prospective cohort studies [20].

In the present study, we used the same pooled data as Tomita et al. [21], but instead of placing all patients in one big group, we performed a post hoc analysis to compare the safety and efficacy of SEMS placement as a BTS for obstructive CRC in CROSS 0 with those in CROSS 1 and 2. As mentioned above, patients classified as CROSS 0 were clinically in a more critical state than those classified as CROSS 1 and 2. Contrary to expectations, both groups showed a high clinical success rate (98.0% vs. 98.4%) as well as a high technical success rate (96.7% vs. 97.8%). Thus, SEMS placement as a BTS for obstructive CRC had good efficacy and high safety in both CROSS 0 and CROSS 1 and 2 patients. Interestingly, complete obstruction was found to be associated with a favorable outcome in terms of the clinical success of colonic stenting.

One particularly important point concerning the present study was our speculation of the cause of the difference in the outcomes of colonic stent procedures between the two groups (complete cases vs. incomplete cases). Unfortunately, we did not evaluate how the bowel was prepared during the procedure in this study. Regarding the visibility of the obstruction site during colonoscopy, it is higher if patients have a complete obstruction as the feces flow from the proximal side to the distal side at the obstruction site is almost entirely cut off in complete obstruction cases. On the other hand, in incomplete obstruction cases, the flow of feces will impair the visibility.

Our ongoing phase III randomized control trial, called the COBRA trial, seeks to verify whether or not decompression with colonic stenting for obstructive CRC is non-inferior to surgery in terms of the disease-free survival (DFS) of stage II/III CRC at three years after primary tumor resection. However, only obstructive CRC patients in CROSS 1 and 2 categories are included in that trial. CROSS 0 patients were excluded because emergency surgery is usually needed in those patients. Given their poor physical status and life-threatening condition, performing primary anastomosis in such patients is considered dangerous. Acute resection for obstructive left-sided colon cancer in an emergency setting results in a high colostomy rate (61.4%) and high risk of conversion to a more invasive approach, such as open surgery (90.8%) [22]. Consequently, the high colostomy (often permanent type) rate decreases the quality of life of patients [23]. We previously reported a high primary anastomosis rate (92%) in a prospective clinical trial on colonic stenting as BTS for obstructive CRC [20]. Taken together, these present and previous findings suggest that it is difficult to obtain informed consent from CROSS 0 patients in this regard.

In addition, stent insertion for obstructive CRC may result in an increase in CK20 mRNA [21], cell-free DNA, circulating tumor DNA [24], and viable circulating tumor cells (v-CTCs) [25]. To accurately evaluate the effect of tumor manipulation by the radial force generated when a SEMS expands, the influence of additional surgery, such as colostomy, should be avoided as much as possible. However, excluding CROSS 0 patients (who are expected to have severe strictures) may reduce the number of cases with adverse events caused by colonic stenting, resulting in an overestimation of the effectiveness and safety of stenting compared to surgery. Therefore, the current study compared the effectiveness and safety of SEMS placement in the CROSS 1 or 2 group and CROSS 0 group.

A longer stricture length and fewer laparoscopic surgeries may be expected in the CROSS 0 group than in the CROSS 1 or 2 group; however, we found that the mean stricture length was 0.6 cm shorter in the CROSS 0 group, and the percentage of laparoscopic surgeries was 4.5% higher. We suspect that this was because of the high number of CRC cases classified as macroscopic type with wall stricture sign (also known as stricture-type CRC) among these patients. Stricture-type CRC looks like a “bow tie” owing to the marked fold convergence of the intestinal tract, causing more than 30% wall shrinkage [26]. Boku et al. showed that the stricture was caused by a high amount of intestinal fibrosis, which might lead to cancer invasion [27]. Despite being smaller than other types, stricture-type CRC can be easily spotted and resected by laparoscopic surgery because of its unique shape. These findings suggest that patients in the CROSS 0 group probably had this type of tumor. Other studies have also found that stricture-type CRC had a considerably higher recurrence rate and shorter recurrence period in the colorectum than other types.

The conversion rate of laparoscopic surgery was 10% in the CROSS 0 group and 4.4% in the CROSS 1 or 2 group. The results of the JCOG 0404 study [28] showed that laparoscopic surgery had a low conversion rate (5.4%) in Japan; however, the CROSS 0 group tended to show a higher rate than the CROSS 1 or 2 groups. The conversion rate of laparoscopic surgery was higher than the average in Japan because obstructive CRC cases were considered. Laparoscopic surgery is more difficult in cases with obstruction than in those without due to inflammation, strong adhesion, and handling of edematous intestine. The CROSS 0 group may require more careful selection for laparoscopic surgery. Park et al. [29] reported that the conversion rate of laparoscopic surgery was 35.3% in the emergency surgery group and 4.3% in the SEMS group; the CROSS 1 or 2 group showed similar but lower results for both conversion rates.

Several limitations associated with the present study warrant mention. First, it was a a non-comparative study with no control group. Furthermore, the long-term outcomes were not studied, and the study was confined to Japan.

In conclusion, two large multicenter, prospective studies demonstrated the short-term efficacy and safety of SEMS placement as a BTS for patients with obstructive CRC classified as CROSS 0, 1, and 2. Furthermore, the effectiveness and safety of SEMS placement as a BTS in the CROSS 0 group were shown to be comparable to those in the CROSS 1 or 2 group.

## Abbreviations

ASA: American Society of Anesthesiologists
BTS: Bridge to surgery
COBRA: Colonic stent for "Bridge to Surgery" prospective randomized controlled trial comparing treatment with non-stenting surgery in stage II/III obstructive colon cancer
CRC: Colorectal cancer
CROSS: ColoRectal Obstruction Scoring System
DFS: Disease-free survival
ECM: Extra-colonic malignancy
ECOG: Eastern Cooperative Oncology Group
ESGE: European Society of Gastrointestinal Endoscopy
JCSSPRG: Japan Colonic Stent Safety Procedure Research Group
PS: Performance status
RCT: Randomized controlled trials
SD: Standard deviations
SEMS: Self-expandable metallic stents
UMIN: University Hospital Medical Information Network
